# The effect of bone marrow-derived mesenchymal stem cell co-transplantation with hematopoietic stem cells on liver fibrosis alleviation and survival in patients with class III β-thalassemia major

**DOI:** 10.1186/s13287-021-02242-8

**Published:** 2021-03-29

**Authors:** Tahereh Rostami, Amir Kasaeian, Nasrollah Maleki, Mohsen Nikbakht, Azadeh Kiumarsi, Seyed Mohammad Tavangar, Amir Pejman Hashemi Taheri, Seied Asadollah Mousavi, Ardeshir Ghavamzadeh

**Affiliations:** 1 Research Institute for Oncology, Hematology and Cell Therapy, Shariati Hospital, Tehran University of Medical Sciences, Kargar Shomali Street, Tehran, 1411713131 Iran; 2 Department of Pathology, Shariati Hospital, Tehran University of Medical Sciences, Tehran, Iran; 3 Department of Radiology, Shariati Hospital, Tehran University of Medical Sciences, Tehran, Iran

**Keywords:** Hematopoietic stem cells, Mesenchymal stem cells, Beta-thalassemia, Liver fibrosis, Outcome

## Abstract

**Background:**

Hepatic fibrosis is a common complication in transfusion-dependent thalassemia patients. Data on the co-transplantation of mesenchymal stem cells (MSCs) with hematopoietic stem cells (HSCs) in beta-thalassemia major patients are scarce. Therefore, we aimed to evaluate the effect of co-transplantation of bone marrow-derived MSC with HSCs on the liver fibrosis alleviation and transplant outcomes in class III beta-thalassemia major.

**Methods:**

Between April 1998 and January 2017, a total of 224 consecutive patients with class III beta-thalassemia major underwent allogeneic HSCT in the Research Institute for Oncology, Hematology and Cell Therapy, Tehran University of Medical Sciences, Tehran, Iran. To assess liver fibrotic changes after transplantation, 47 patients participated in the MSC plus HSC group and 30 patients in the HSC only group at the end of the follow-up period. All patients underwent laboratory tests, especially serum ferritin and liver function testing, hepatic T2* MRI, liver biopsy, and FibroScan before and 2 years after transplantation. Kaplan-Meier curves were derived to determine survival and were compared using the log-rank test. Repeated-measure, mixed-effect linear regression models were used to examine the changes in liver fibrosis over time.

**Results:**

The 10-year OS rate was 71.84% in the mesenchymal group and 61.89% in the non-mesenchymal group (*P* value = 0.294), while the 10-year TFS rate was 63.64% in the mesenchymal group and 52.78% in the non-mesenchymal group (*P* value = 0.285). No significant difference was observed in the 10-year NRM, rejection rate, ANC engraftment, platelet engraftment, acute GvHD, and chronic GvHD between the two groups. In addition, the results of repeated-measure, mixed-effect linear regression models showed that none of the variables determining hepatic fibrosis had a significant difference between patients receiving MSCs and patients who did not receive MSCs.

**Conclusions:**

Based on the results of this study, a single infusion of MSCs at the time of HSCT to patients with class III beta-thalassemia major could not significantly improve the liver fibrosis alleviation and transplantation outcomes, including OS, TFS, TRM, rejection rate, ANC engraftment, platelet engraftment, acute GvHD, and chronic GvHD.

**Supplementary Information:**

The online version contains supplementary material available at 10.1186/s13287-021-02242-8.

## Introduction

Thalassemia major (TM) is one of the most common inherited genetic hematological diseases worldwide and is associated with significant morbidity and mortality rates [1, 2]. One of the most common clinical problems in patients with transfusion-dependent thalassemia major (TDTM) is iron overload in various organs such as the liver, heart, pancreas, endocrine glands, lungs, and kidneys [3, 4]. Based on the current guidelines, allogeneic hematopoietic stem cell transplantation (HSCT) remains the only curative treatment option for eligible TM patients and can improve long-term survival [5–9]. It is reported that more than 90% of patients with thalassemia major survive after HSCT and disease-free survival (DFS) is about 80% [6].

Hepatic fibrosis is a common complication in transfusion-dependent thalassemia patients. In these patients, the liver is the primary site of iron deposition, leading to fibrosis and then cirrhosis [10, 11]. Among patients with TDTM, the prevalence of liver fibrosis and cirrhosis has been reported to be 40–80% and 10–40%, respectively [12–14]. It seems that hepatic fibrosis in TM patients depends on the patient’s age, liver iron concentration, and also the number of infused packed red blood cell units [15]. Recent evidence has shown that thalassemia patients with moderate to severe liver iron concentrations experience persistently elevated serum iron levels for several years after HSCT [16]. Therefore, they are at increased risk of developing liver fibrosis and cirrhosis after HSCT [17].

Recently, stem cell-based therapy using mesenchymal stem cells (MSCs) has been considered as an interesting treatment option for the reduction of liver fibrosis. These cells are unique and have different and specific functions that include (1) immune-modulatory properties, (2) differentiation into hepatocytes together with antifibrotic effects, (3) potential ability to improve regeneration of residual hepatocytes, (4) inhibition of hepatic stellate cell activation and subsequent replacement of injured hepatocytes, and (5) growth factors and cytokine secretion which promote hepatic regeneration [18–23]. To date, several studies have been performed to evaluate the clinical therapeutic effects of MSC transplantation in liver cirrhosis [24–32]. However, with a short-term follow-up, most of these studies had conflicting results on the improvement of liver function.

Moreover, the in vivo ability of hepatocytes differentiated from MSCs during co-transplantation of MSCs and hematopoietic stem cells (HSCs) to regenerate the damaged hepatocytes in thalassemia major patients with chronic liver disease has not been clearly demonstrated yet. Furthermore, there has not been any prospective cohort study with long-term follow-up evaluating the efficacy of co-transplantation of bone marrow-derived MSCs with HSCs in thalassemia major patients. Therefore, we conducted this prospective cohort study with long-term follow-up in patients with Lucarelli risk classification (LRC) class III β-thalassemia major to evaluate the effect of co-transplantation of bone marrow-derived MSCs with HSCs on the liver fibrosis alleviation as well as post-transplant outcome in this setting.

## Materials and methods

### Study design and population

Between April 1998 and January 2017, a total of 248 consecutive patients with LRC class III β-thalassemia major underwent allogeneic HSCT in the Research Institute for Oncology, Hematology and Cell Therapy (RIOHCT), affiliated to Tehran University of Medical Sciences, and were enrolled in this single-center prospective randomized controlled trial. Out of these patients, 24 were excluded from the study due to cardiovascular disease (*n* = 7), renal failure (*n* = 3), uncontrolled diabetes mellitus (*n* = 5), and viral hepatitis (*n* = 9). Finally, 224 eligible patients were randomly assigned to receive co-transplantation of bone marrow-derived MSCs with HSCs (*n* = 83) or to receive HSCs alone (*n* = 131).

**Fig. 1:**
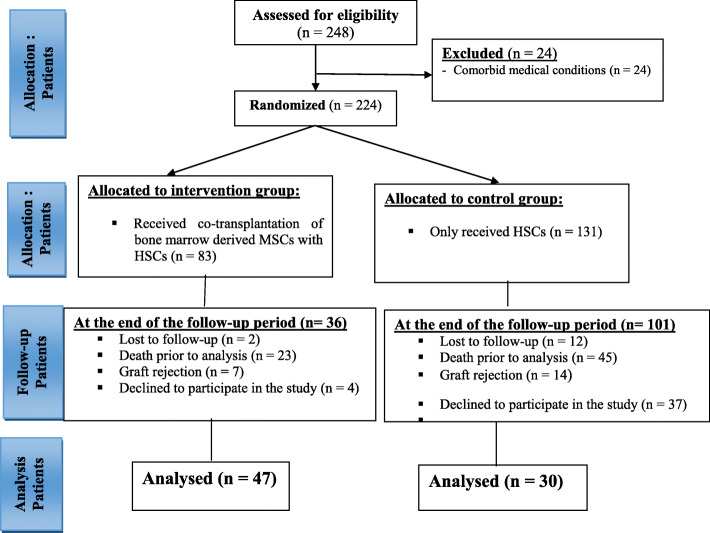
Flow diagram of participants throughout the study

In order to assess liver fibrotic changes after HSCT, in the second phase of the study, 47 patients participated in the MSC plus HSC group and 30 patients in the HSC only group (Fig. 1). In this phase, participants who met any of the following criteria were excluded from the study: (1) patients who declined to participate in the study, (2) any severe underlying or pre-existing medical condition other than thalassemia major and its obvious complications, (3) those who lost a regular follow-up visit, (4) patients who did not gain complete hematological recovery after transplantation, (5) patients who had received drugs with hepatic metabolism 6 months before the study, and (6) patients with class I and II thalassemia major.

Hemoglobin electrophoresis and mutation analyses were performed on all patients to confirm the diagnosis of β-thalassemia major. Patients were classified using the LRC before HSCT based on the evidence of portal fibrosis in liver biopsy, the presence of hepatomegaly (liver size greater than 2 cm palpable below the right costal margin), and inadequate chelation therapy. Patients with none of the above risk factors were classified as class I, patients with 1 or 2 of these risk factors were classified as class II, and patients with all three risk factors were classified as class III [33, 34].

The Ethics Committee of Tehran University of Medical Sciences approved the study (Ethical code: IR.TUMS.MEDICINE.REC.1398.093, research code: 97-02-36-38570), and the study was conducted under the Declaration of Helsinki. Written informed consent was obtained from all participants and their parents.

### Evaluation of hepatic fibrosis

All participants underwent laboratory tests, including serum ferritin level and liver function tests, hepatic T2* magnetic resonance imaging (MRI), liver biopsy, and FibroScan (liver elastography) before and after HSCT.

### Hepatic T2* MRI

Hepatic T2* MRI was performed in the Department of Radiology using a 1.5-T Magnetom Siemens Symphony scanner (Siemens Medical Solutions, Erlangen, Germany). All the patients were placed in a supine position and entered the magnet cradle, using the head-first configuration. A 10-mm slide thickness through the liver core scanned at 12 different echo times (TE 1.3 to 23 ms) was used for the calculation of hepatic T2 values. The repetition time (TR) was 200 ms, the base resolution matrix was 128 pixels, the field of view was 39.7 cm × 19.7 cm, the flip angle used was 20°, and the sampling bandwidth was 125 kHz. Each image was taken within 11–13 s of breath-holding using a gradient-echo sequence. T2* values were calculated by the software (CMR tools, Imperial College). The results of hepatic T2* were categorized as normal (T2* > 30 ms), mild (6.2 < T2* < 30 ms), moderate (3.1 < T2* < 6.2 ms), severe (2.1 < T2* < 3.1 ms), and very severe (T2* < 2.1 ms) [35].

### FibroScan (transient elastography)

All patients were assessed by transient elastography (FibroScan®, EchoSens, Paris, France) on the same day as the liver biopsy examination. This method was performed by a skilled gastroenterologist who was blind to clinical and histological data. Measurements were taken in the right lobe of the liver, through intercostal spaces while the patient was lying in dorsal decubitus, with the right arm at maximal abduction. Using ultrasound, a portion of the liver at least 6 cm thickness and free of large vessels was recognized for examination. Only procedures were considered reliable that at least ten validated measurements were performed on each patient; the success rate was at least 60%, and an interquartile range (IQR) of the median stiffness value was lower than 30% [36]. The results were expressed in kilopascals (kPa). A cutoff value less than 7.0 kPa was not considered significant for liver fibrosis [37].

### Histological data

Liver biopsy was performed by an expert radiologist with an 18-gauge (18-G) needle (Bard Peripheral Vascular, Biopsy System Max Core®, USA) under ultrasound guidance. Liver biopsy specimens were fixed in formalin and embedded in paraffin. The sections of liver tissue were stained with hematoxylin-eosin and Masson trichrome and read by one experienced liver pathologist blinded to the results of liver transient elastography and T2* MRI. The METAVIR scoring system was used for the assessment of liver fibrosis and necro-inflammatory activity [38]. Fibrosis was staged on a 0 to 4 scale as follows: F0, no fibrosis; F1, portal fibrosis without septa; F2, periportal fibrosis and few septa; F3, bridging fibrosis and numerous septa without cirrhosis; and F4, cirrhosis. Activity was graded as follows: A0, none; A1, mild; A2, moderate; and A3, severe.

### Isolation of mesenchymal stem cells

All patients received MSC and HSC from the same donor. For the generation of MSCs, about 40–90 mL of bone marrow aspirate was obtained from HLA-matched siblings or other HLA-matched relative donors before bone marrow transplantation.

Briefly, bone marrow mononuclear cells (MNCs) were then separated using Ficoll-Paque (Stem Cell Technologies, Vancouver, Canada) (400 g, 25 min, 20 °C) and washed twice by phosphate-buffered saline (PBS). Mononuclear cells were seeded at a density of 160 × 10^3^ cells per cm^2^ and cultured in a complete culture medium consisting of the following: Dulbecco’s modified Eagle’s medium-low glucose (DMEM; Gibco, Life Technologies, USA), supplemented with 10% fetal bovine serum (FBS; Gibco, Life Technologies, USA), and 1% (vol/vol) penicillin/streptomycin (Gibco, Life Technologies, USA) with final concentrations of 100 units/mL and 100 μg/mL, respectively, was irradiated with a dose of 25 kGy. Cultures were maintained in a humidified atmosphere containing 5% CO_2_ at 37°. Cells were allowed to adhere for 48 h; henceforth, non-adherent cells were discarded, and the culture medium was replaced twice weekly. After reaching ≥70% confluence, MSCs were harvested using trypsin/EDTA (Gibco, Life Technologies, USA) and re-plated at 4000 cells per cm^2^. After the final harvesting (usually at passage 2–3), MSCs were cryopreserved with 10% (v/v) dimethyl sulfoxide (DMSO) with USP grade components (Miltenyi Biotec, Germany) and 5% human albumin (Biotest AG, Germany) until the time of bone marrow transplantation. The collected cells were identified to meet the minimal criteria of MSCs according to the International Society of Cell Therapy (ISCT) standard. The quality and viability of these cells were reconfirmed after preparation and before each infusion for the patient. MSCs are characterized by the following criteria: fibroblast-like morphology; the ability to self-renew; expression of cell surface markers including CD73, CD105, CD90, and HLA-DR; and no expression of CD45, CD34, CD14, CD11b, CD79, or CD19. Additionally, MSCs must be capable of in vitro differentiation into osteoblasts, adipocytes, and chondrocytes.

The median MSC dose was 2.0 × 10^6^ cells/kg of the recipient’s body weight. The total volume of the MSC infusion was 50 mL and infused 3 h prior to HSCT.

### Stem cell source, donor selection, conditioning regimen, and GVHD prophylaxis

The sources of stem cells were bone marrow stem cells (BMSCs) and peripheral blood stem cells (PBSCs). In bone marrow cases, donor stem cells were harvested from the posterior iliac crest under general anesthesia. Mobilization of PBSCs was performed by the administration of 5 μg/kg/day granulocyte-colony stimulating factor (G-CSF) 4 consecutive days and 5 μg/kg twice daily for the fifth day and then collected using a continuous-flow leukapheresis.

We administered a myeloablative conditioning regimen for all patients, consisted of intravenous busulfan at 3.5 mg/kg/day from day − 9 for 4 consecutive days and cyclophosphamide at 40 mg/kg/day from day − 5 for 4 consecutive days. For HSCT recipients other than HLA identical sibling (HLA-matched unrelated and cord blood recipients), in addition to the mentioned regimen, rabbit ATG (Thymoglobulin; Sanofi-Aventis, Quebec, Canada) was given intravenously at a dose of 2.5 mg/kg/day from days − 5 to − 2.

Regarding GvHD prophylaxis, cyclosporine (1.5 mg/kg/day, IV, on day − 2, and then 3 mg/kg/day on day + 7 in PBSC and day + 11 in BMSC) in combination with a short course of methotrexate (10 mg/m^2^ on days + 1 and 6 mg/m^2^ on days + 3,+ 6, and + 11) was administered for patients. Cyclosporine was converted to oral formulation when patients were able to tolerate oral intake. It was continued orally for at least 6 months after HSCT and discontinued in the absence of graft-versus-host disease (GVHD).

**Fig. 2:**
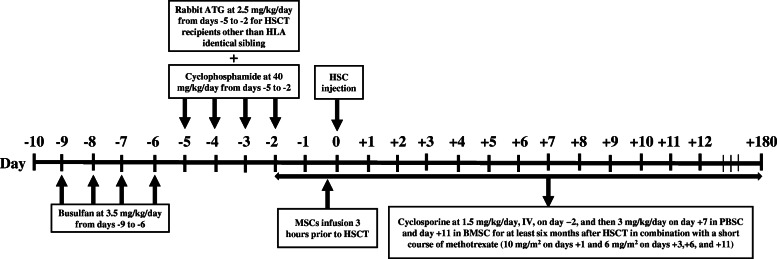
Diagram of therapeutic intervention around the HSCT in the time of co-transplantation of the BM-derived MSCs

The diagram of the therapeutic intervention around the HSCT in the time of co-transplantation of the BM-derived MSCs is shown in Fig. 2.

### Definitions and outcome measurements

Neutrophil engraftment was defined as the first date for 3 consecutive days with an absolute neutrophil count of more than 0.5 × 10^9^/L without G-CSF subcutaneous injection, and platelet engraftment was defined as the first date for 7 consecutive days with a platelet count of greater than 20,000 unsupported by platelet transfusions. Full chimerism was assumed when > 95% of the recipient’s blood cells were from the donor’s origin. When this index fell below 5%, graft rejection was considered. Any rate between the two above was defined as mixed chimerism. Standard criteria were considered to define and diagnose acute and chronic GVHD [39, 40].

The primary endpoint was the evaluation of hepatic fibrotic changes as measured by FibroScan score, hepatic T2* MRI, and liver histology. The secondary endpoints were overall survival (OS), thalassemia-free survival (TFS), non-relapse mortality (NRM), and GVHD.

### Statistical analysis

Homogeneity between the two groups of patients was evaluated using the chi-square test for qualitative variables and median test and Student’s *t* test for continuous variables. A two-sided *P* value of 0.05 or lower was considered to be statistically significant. Kaplan-Meier curves were derived to determine OS and TFS and were compared using the log-rank test. The median follow-up time was established with the reverse Kaplan-Meier method. The assumption of proportionality of hazards was checked using Schoenfeld residuals. Analyses were conducted using STATA version 11.2. The linear mixed model was used for the repeated measurement data from the follow-up visits.

Repeated-measure, mixed-effect linear regression models [41] were used to examine the change over time in serum ferritin, aspartate aminotransferase (AST), alanine aminotransferase (ALT), FibroScan score, hepatic T2* MRI, liver stage, liver grade, and dry weight iron concentration. Evaluation of the intervention effect as a change over time in each of the variables among all patients (model 1) and determination of time-by-mesenchyme interaction in each variable (model 2) was conducted. All models included a variable indexing time (pre-HSCT and post-HSCT), dichotomous indicators of intervention (with mesenchymal vs without mesenchymal), and baseline fixed effects for participant characteristics (i.e., patient’s age, sex matching, ABO matching, source of HSCT, acute GVHD, and chronic GVHD). In addition, we used post hoc estimation to disaggregate two-way interaction and generate model-based tools and standard errors, and we used *t* tests to evaluate the simple main effects.

## Results

### Characteristics of all patients

A total of 214 patients were enrolled in the study, including 83 (38.8%) patients who underwent co-transplantation of MSCs with HSCs (mesenchymal group) and 131(61.2%) patients who received only HSCs (non-mesenchymal group). The median age of patients in the mesenchymal group was 15.4 years (range, 11.8–19 years) compared with 12.9 years (range, 9.2–18.4 years) in the non-mesenchymal group. Characteristics of all patients are shown in Table 1. The mortality etiologies included GVHD, sepsis, and graft rejection.

**Table 1:** Baseline patient demographic and disease characteristics of the study population

Characteristics		Mesenchymal patients	Non-mesenchymal patients	*P* value
Number (%)		83	131	
Age (years), median (P_25_–P_75_)		15.37 (11.77–19)	12.89 (9.22–18.4)	0.023
Patient sex, *n* (%)	Male	54 (65.1%)	74 (56.5%)	0.213
	Female	29 (34.9%)	57 (43.5%)	
Donor sex, *n* (%)	Male	41 (51.3%)	51 (42.9%)	0.244
	Female	39 (48.7%)	68 (57.1%)	
Sex match status, *n* (%)	Sex-mismatched	48 (60%)	54 (45.4%)	0.043
	Sex-matched	32 (40%)	65 (54.6%)	
ABO match status, *n* (%)	ABO-mismatched	30 (37.5%)	46 (37.4%)	0.988
	ABO-matched	50 (62.5%)	77 (62.6%)	
Survival status, *n* (%)	Alive	60 (72.3%)	86 (65.6%)	0.309
	Dead	23 (27.7%)	45 (34.4%)	
Source of HSCT, *n* (%)	BM	29 (34.9%)	52 (39.7%)	0.485
	PB	54 (65.1%)	79 (60.3%)	
Acute GvHD, *n* (%)	No	40 (48.2%)	74 (56.5%)	0.236
	Yes	43 (51.8%)	57 (43.5%)	
Chronic GvHD, *n* (%)	No	53 (63.9%)	81 (61.8%)	0.766
	Yes	30 (36.1%)	50 (38.2%)	
Stem cell number in grafts	WBC (× 10^8^/kg), median (P_25_–P_75_)	8.12 (5.79–10.05)	8.41 (6.27–9.32)	0.758
	MNC (× 10^8^/kg), median (P_25_–P_75_)	7.03 (3–8.07)	6.80 (3.5–8.36)	0.317
	CD_3_ cell (× 10^6^/kg), median (P_25_–P_75_)	213.22 (53–339)	221.05 (75.8–314.8)	0.836
	CD34 cell (× 10^6^/kg), median (P_25_–P_75_)	3.9 (2.5–6.1)	3.4 (2.5–7.4)	0.712

*GVHD* graft-versus-host disease, *HSCT* hematopoietic stem cell transplantation, *MNC* mononuclear cell, *WBC* white blood cells, *BM* bone marrow, *PB* peripheral blood

### Post-HSCT outcomes

At the median follow-up time of 7.2 years (range, 3.9–18.8 years) from transplantation, 68 (30.4%) of the patients were dead. The number of patients alive at the last follow-up between the mesenchymal and non-mesenchymal groups was 60 (72.3%) and 86 (65.6%) (*P* = 0.309), respectively.

As shown in Table 2, the 10-year OS rate was 71.84% in the mesenchymal group and 61.89% in the non-mesenchymal group. Although the 10-year OS was approximately 10% higher in the mesenchymal group than in the non-mesenchymal group, this difference was not statistically significant (*P* value = 0.294). Furthermore, the 10-year TFS rate was 63.64% in the mesenchymal group and 52.78% in the non-mesenchymal group (*P* value = 0.285). In this study, we could not find any statistically significant relationship between the 10-year OS and 10-year TFS and other studied variables (Table 2). As shown in Table 3, no significant difference was observed in the 10-year NRM and rejection rate between the two groups.

**Table 2:** The 10-year overall survival (OS) and thalassemia-free survival (TFS) in the study population

All patients		OS (95% CI)	^●^*P* value	TFS (95% CI)	^●^*P* value
Mesenchymal group	No	61.89 (51.61–70.60)	0.294	52.78 (42.78–61.82)	0.285
	Yes	71.84 (60.71–80.32)		63.64 (52.26–72.99)	
Patient sex	Female	72.09 (6088–8059)	0.131	67.94 (56.79–76.79)	0.245
	Male	61.06 (51.04–69.63)		59.35 (349.52–58.44)	
Donor sex	Female	66.37 (54.58–75.77)	0.561	56.63 (45.15–66.59)	0.999
	Male	65.78 (55.18–74.45)		57.66 (47.02–66.91)	
Sex-matched	Mismatched	62.57 (50.93–72.19)	0.458	50.76 (39.58–60.90)	0.098
	Matched	68.72 (58.29–77.05)		62.93 (52.49–71.69)	
ABO-matched	ABO-matched	68.36 (58.15–76.58)	0.248	60.49 (50.36–69.19)	0.158
	ABO-mismatched	62.17 (50.43–71.90)		51.65 (40.17–61.97)	
Source of transplant	BM	72.82 (61.29–81.43)	0.194	61.96 (50.18–71.73)	0.492
	PB	62.21 (52.58–70.43)		54.45 (44.95–63.01)	
Acute GvHD	No	73.09 (62.21–81.30)	0.490	58.17 (47.36–67.51)	0.594
	Yes	67.64 (59.42–71.51)		56.63 (46.41–65.62)	
Chronic GvHD	No	64.29 (58.13–70.17)	0.867	68.02 (59.27–76.23)	0.341
	Yes	68.46 (59.19–76.39)		73.46 (61.19–82.39)	

*OS* overall survival, *TFS* thalassemia-free survival, *GvHD* graft-versus-host disease, *BM* bone marrow, *PB* peripheral blood

^●^Log-rank test

**Table 3:** The 10-year non-relapse mortality (NRM) and rejection rate in the study population

Groups		Rejection rate	*P* value	Non-relapse mortality (NRM)	*P* value
		10 years		10 years	
Mesenchymal group	Yes	14.46% (7.89–22.93%)	0.88	22.02% (13.69–31.59%)	0.222
	No	15.52% (9.87–22.34%)		31.88% (22.66–41.47%)	
Sex-matched	Mismatched	18.52% (11.66–26.61%)	0.08	31.20% (20.90–42.01%)	0.756
	Matched	10.23% (5.21–17.23%)		26.74% (18.35–35.85%)	
ABO-mismatched	Mismatched	18.32% (10.78–27.43%)	0.236	30.13% (20.32–40.53%)	0.543
	Matched	12.53% (7.48–18.94%)		27.48% (18.50–37.21%)	
Source of HSCT	PB	13.68% (8.45–20.17%)	0.404	32.12% (23.46–41.09%)	0.109
	BM	17.39% (10.01–26.46%)		20.68% (12.32–30.55%)	
Acute GvHD	Yes	15.95% (12.42–21.77%)	0.452	37.47% (28.06–46.85%)	0.256
	No	22.86% (15.73–30.80%)		29.31% (21.10–39.21%)	
Chronic GvHD	Yes	16.82% (12.46–25.31%)	0.249	26.77% (16.58–38.02%)	0.349
	No	24.32% (17.44–31.83%)		27.70% (20.27–35.60%)	

*TRM* transplant-related mortality, *GvHD* graft-versus-host disease, *BM* bone marrow, *PB* peripheral blood

The median time to neutrophil engraftment for patients in mesenchymal and non-mesenchymal groups was 16.8 (range 11–42) and 18.1 (range 12–51) days, respectively (*P* = 0.764), and platelet engraftment was 19.8 (range 12–51) and 25.6 (range 14–58) days, respectively (*P* = 0.273). There were no significant differences observed in the cumulative incidence of neutrophil (*P* = 0.450) and platelet (*P* = 0.299) engraftment between the mesenchymal and non-mesenchymal groups.

Moreover, for patients in the mesenchymal and non-mesenchymal groups, grade II–IV acute GVHD was observed in 43 (51.8%) and 57 (43.5%) patients, respectively (*P* = 0.236), while moderate/severe chronic GVHD was seen in 30 (36.1%) vs 50 (38.2%) patients, respectively (*P* = 0.766). There were no significant differences seen in the cumulative incidence of acute GVHD (*P* = 0.490) and chronic GVHD (*P* = 0.866) between mesenchymal and non-mesenchymal groups.

**Fig. 3:**
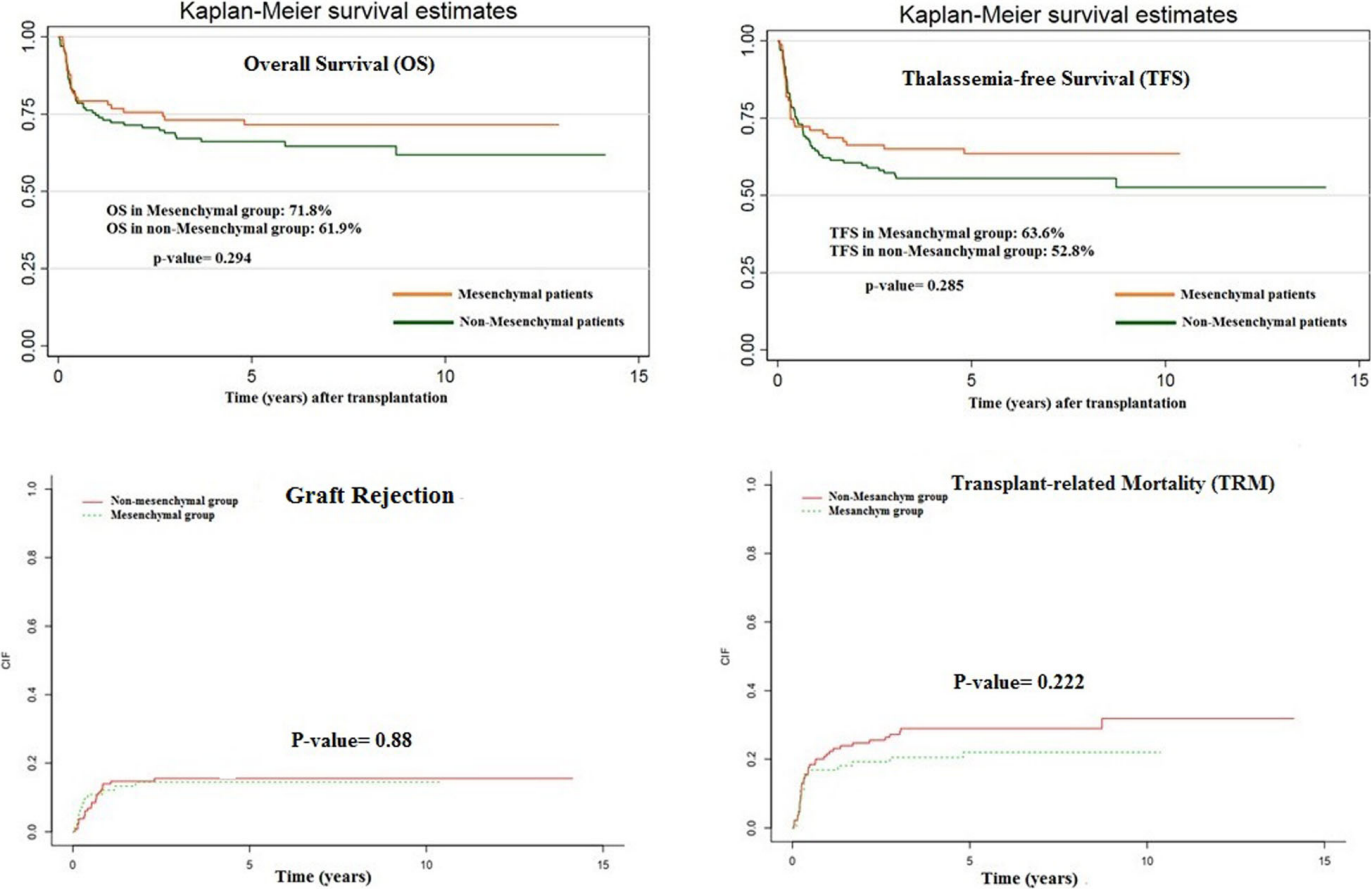
Kaplan-Meier curves for overall survival (OS), thalassemia-free survival (TFS), transplant-related mortality (TRM), and graft rejection rate

Data on the 10-year transplant outcomes according to the source of HSCT in the study population showed that the BM + MSC group has a better OS and TFS compared to the other groups, although this difference was not statistically significant (*P* value = 0.402). Kaplan-Meier curves for OS, TFS, NRM, and graft rejection rate are shown in Fig. 3.

### Characteristics of patients who underwent liver fibrosis evaluation

To evaluate liver fibrosis, a total of 77 patients were enrolled, comprising 47 patients in the mesenchymal group and 30 patients in the non-mesenchymal group. The median age of patients in the mesenchymal group was 15.4 years (range, 10.9 to 16.8 years) compared with 11.5 years (range, 5.7 to 15.1 years) in the non-mesenchymal group. Patient characteristics of the study population are shown in Table 4. For patients in mesenchymal and non-mesenchymal groups, grade II–IV acute GVHD was observed in 11 (23.4%) and 8 (26.7%) patients, respectively (*P* = 0.316), whereas moderate to severe chronic GVHD was seen in 7 (14.9%) vs 5 (16.7%) patients, respectively (*P* = 0.464).

**Table 4:** Baseline patient demographic and disease characteristics in patients who evaluated for liver fibrosis

Characteristics		Mesenchymal patients (*n* = 47, 61%)	Non-mesenchymal patients (*n* = 30, 39%)	*P* value
Age (years), mean (P_25_–P_75_)		15.4 (10.94–16.81)	11.5 (5.73–15.13)	0.019
Patient sex, *n* (%)	Male	27 (57.5%)	10 (33.3%)	0.039
	Female	20 (42.5%)	20 (66.7%)	
Donor sex, *n* (%)	Male	25 (53.2%)	10 (38.5%)	0.228
	Female	22 (46.8%)	16 (61.5%)	
Sex match status, *n* (%)	Sex-mismatch	26 (55.3%)	10 (38.5%)	0.168
	Sex-match	21 (44.7%)	16 (61.5%)	
ABO match status, *n* (%)	ABO-mismatched	18 (39.1%)	7 (25%)	0.213
	ABO-matched	28 (60.9%)	21 (75%)	
Source of HSCT, *n* (%)	BM	14 (29.8%)	14 (46.7%)	0.133
	PB	33 (70.2%)	16 (53.3%)	
Acute GvHD, *n* (%)	No	21 (44.7%)	17 (56.7%)	0.305
	Yes	26 (55.3%)	13 (43.3%)	
Chronic GvHD, *n* (%)	No	24 (51%)	16 (53.3%)	0.846
	Yes	23 (49%)	14 (46.7%)	
WBC (× 10^8^/kg), mean (P_25_–P_75_)		8.38 (6.07–10.24)	7.13 (5.7–9.67)	0.732
MNC (× 10^8^/kg), mean (P_25_–P_75_)		7.41 (3.3–8.11)	4.35 (2.71–8.22)	0.427
CD_3_ cell (× 10^6^/kg), mean (P_25_–P_75_)		236.8 (76.02–335.6)	83.86 (62.3–269)	0.126
CD34 cell (× 10^6^/kg), mean (P_25_–P_75_)		4.23 (2.6–6.42)	3.59 (2.8–8.07)	0.837
ANC engraftment (days), mean (P_25_–P_75_)		16.8 (14–21)	18.1 (15–24)	0.764
Platelets engraftment (days), mean (P_25_–P_75_)		25.8 (21–32)	35.6 (24–51)	0.273

### Evaluation of liver fibrosis

**Fig. 4:**
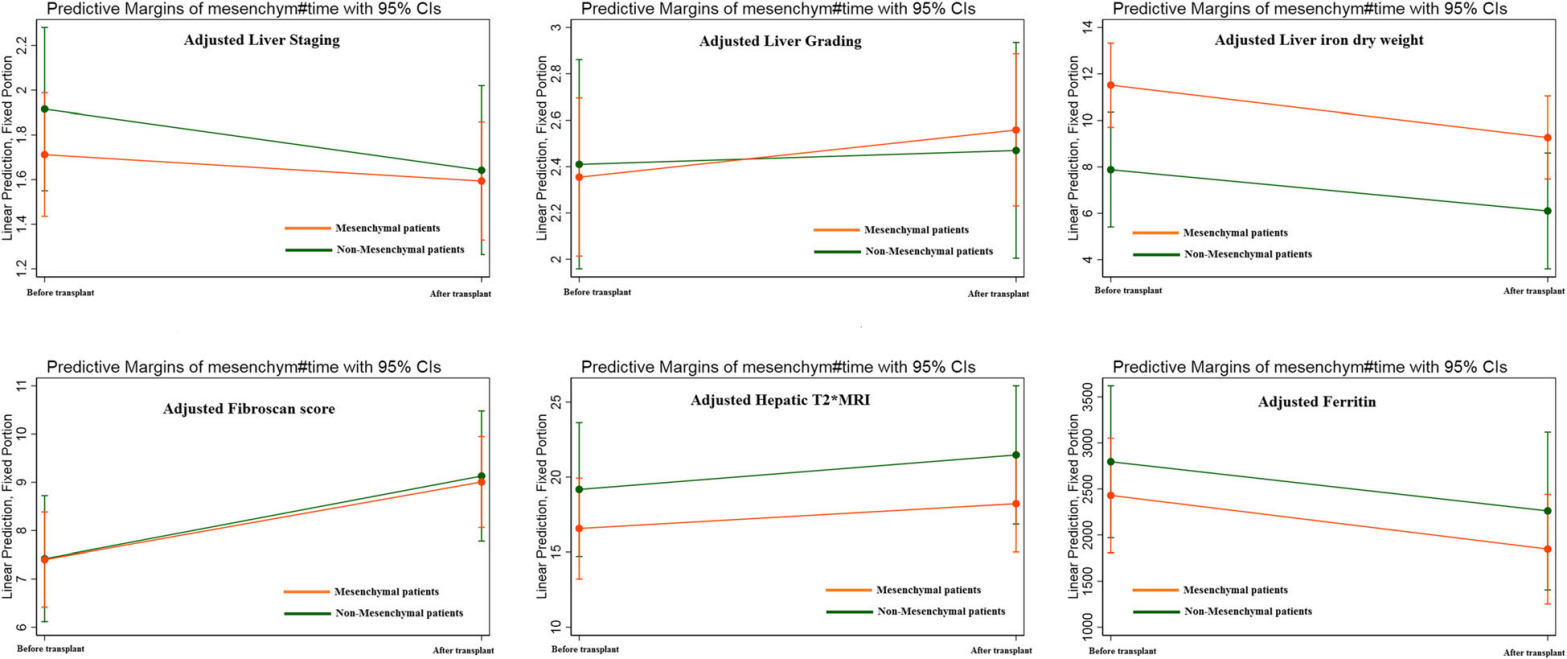
Adjusted figures of each of the variables before and after the HSCT in the study population

For liver fibrosis evaluation, we compared the following variables before and after HSCT between the two groups: serum ferritin, AST, ALT, FibroScan score, hepatic T2* MRI, liver grading and staging based on histological features, and liver iron concentration (LIC). Repeated-measure, mixed-effect linear regression models were used to examine the change over time on these variables. The results showed that none of the studied variables had a significant difference between patients receiving MSCs and patients who did not receive MSCs (Table 5). This means that MSC co-transplantation cannot alleviate liver fibrosis in patients with class III β-thalassemia Major. Adjusted figures of each of the variables before and after the HSCT are given in Fig. 4. Also, model fit statistics are presented in Supplement 1.

**Table 5:** Model-derived means and tests assessing the changes in hepatic fibrosis: the main effects and interactions

Model	Variable	Pre-HSCT		Post-HSCT		Δ (se)	*P* value
Model 1: Time	Ferritin	2555.47 (239.82)		1995.70 (239.82)		− 559.77 (339.15)	0.101
	AST	29.06 (1.169)		29.04 (1.169)		−0.02 (1.65)	0.99
	ALT	37.67 (1.58)		40.08 (1.58)		2.41 (2.24)	0.282
	FibroScan score	7.40 (0.38)		9.06 (0.38)		1.66 (0.54)	**0.0025**
	Hepatic T2* MRI	19.42 (1.31)		17.43 (1.31)		−1.99 (1.85)	0.283
	Liver stage	1.78 (0.11)		1.62 (0.11)		−0.16 (0.15)	0.297
	Liver grade	2.37 (0.13)		2.53 (0.13)		0.15 (0.19)	0.412
	Liver iron dry weight	10.22 (0.71)		8.17 (0.71)		−2.05 (1.01)	**0.0442**
Model 2: Time*Intervention	Variable	Pre-HSCT		Post-HSCT		Δ (se)	*P* value
		Without mesenchyme	With mesenchyme	Without mesenchyme	With mesenchyme		
	Ferritin	2835.04 (405.78)	2404.04 (308.50)	2305.80 (419.24)	1826.65 (296.44)	48.16^a^ (347.49)	0.89
	AST	29.30 (1.97)	28.90 (1.50)	28.65 (2.03)	29.27 (1.45)	−1.02^a^ (1.92)	0.596
	ALT	37.41 (2.67)	40.20 (2.74)	37.79 (2.02)	40.02 (1.96)	0.55^a^ (2.68)	0.837
	FibroScan score	7.46 (0.64)	7.36 (0.49)	9.19 (0.66)	8.99 (0.47)	−0.10^a^ (0.68)	0.881
	Hepatic T2* MRI	19.55 (2.21)	16.31 (1.67)	21.91 (2.27)	18.04 (1.62)	−0.63^a^ (2.21)	0.775
	Liver stage	1.98 (0.18)	1.66 (0.14)	1.72 (0.19)	1.56 (0.13)	0.16^a^ (0.24)	0.518
	Liver grade	2.42 (0.22)	2.34 (0.17)	2.49 (0.23)	2.55 (0.16)	0.14^a^ (0.36)	0.689
	Liver iron dry weight	8.11 (1.22)	11.37 (0.90)	6.36 (1.23)	9.14 (0.89)	−0.47^a^ (0.93)	0.613

Mixed-effect linear regression models adjusted for patient’s age, sex matching, ABO matching, source of HSCT, aGvHD, cGvHD, ferritin, and dry iron liver

*Se* standard error

^a^ΔD = (post-HSCT-positive mesenchymal − pre-HSCT-positive mesenchymal) − (post-HSCT-negative mesenchymal − pre-HSCT-negative mesenchymal)

## Discussion

Liver fibrosis is a common complication in transfusion-dependent thalassemia patients due to hepatic iron overload and hepatitis virus C infection [42–44]. Cell-based therapy using MSCs has recently been considered as an interesting therapeutic option for the reduction of liver fibrosis. It has been demonstrated that MSCs have beneficial effects in a wide range of clinical settings. They can suppress inflammatory responses, increase hepatocyte regeneration, reduce hepatocyte apoptosis, increase liver function, and regress liver fibrosis [45]. However, the clinical effectiveness and safety of MSC-based therapy in liver disease is still debatable [46]. MSC-based therapy for hepatic fibrosis has been performed mainly in patients with cirrhosis caused by different types of liver disease [24–32].

To date, no studies have investigated the use of bone marrow-derived MSCs for the improvement of liver fibrosis in thalassemia major. As reported in our previous study on a subgroup of thalassemia major patients, the co-transplantation of MSCs and HSCs to class III thalassemia major patients does not alter their transplantation outcomes [47]. In the present study, the co-transplantation of bone marrow-derived MSCs and HSCs in patients with LRC class III beta-thalassemia major could not significantly improve the liver fibrosis alleviation and transplantation outcomes, including OS, TFS, TRM, reject incidence, ANC engraftment, platelet engraftment, acute GvHD, and chronic GvHD.

This clinical information is consistent with experimental results previously obtained using animal models. It has been conclusively shown that the mesenchymal tissue that supports donor type hematopoiesis is of host origin [48–50]. In addition, intravenous transplantation of MSCs from transgenic mice showed that MSCs have a short lifespan after transplantation and do not migrate out of the lungs [51–53].

Based on the in vitro immunomodulatory properties of MSCs, these cells have been used in the treatment of acute GvHD [54]. MSC therapeutic activity is achieved only when MSCs are transfused in the presence of a specific inflammatory substance [55]. According to these data, mesenchymal stromal cells were effective in reducing the symptoms of GvHD only when multiple transfusions were performed after transplantation, but not when a single dose was co-transplant with HSCT [56, 57]. Given that, we used only a single dose of MSCs with HSCT in our study, perhaps this factor explains why there was no significant difference in reducing GVHD incidence in patients who were co-transplanted with MSC and HSC compared to the HSC only transplanted group.

In a previous study, we showed that co-transplantation of HSC with MSCs increases the rate of replacement of recipient hepatocytes by donor-derived cells and may improve liver fibrosis; however, significant improvement in liver fibrosis was not seen in this study [58]. Some researchers believe that MSCs may reduce the risk of graft failure, possibly due to their immunosuppressive effect on alloreactive host T lymphocytes [59, 60]. In addition, it is mentioned that co-transplantation of MSCs and HSCs leads to rapid engraftment of ANC and platelets [61]. However, in our patients, the rates of ANC engraftment, platelet engraftment, and rejection did not significantly differ in the mesenchymal group compared to the non-mesenchymal group.

The main limitations of this study were being a single-centered, limited sample size, and refusal of most patients to undergo liver biopsy after HSCT.

## Conclusion

Based on the results of this study, a single infusion of MSCs at the time of HSCT in patients with class III β-thalassemia major has no beneficial effect on transplantation outcomes and liver fibrosis alleviation, so routine implementation of this expensive procedure is not recommended unless the results of further studies prove otherwise.

## Supplementary Information

**Additional file 1.** Methodological Appendix.

## Data Availability

All data generated or analyzed during this study are included in this article.

## Abbreviations

ALT: Alanine aminotransferase
ANC: Absolute neutrophil count
AST: Aspartate aminotransferase
ATG: Anti-thymocyte globulin
BMSCs: Bone marrow stem cells
DFS: Disease-free survival
GVHD: Graft-versus-host disease
HSCs: Hematopoietic stem cells
HSCT: Hematopoietic stem cell transplantation
LRC: Lucarelli risk classification
MNCs: Mononuclear cells
MRI: Magnetic resonance imaging
MSCs: Mesenchymal stem cells
NRM: Non-relapse mortality
OS: Overall survival
PBSCs: Peripheral blood stem cells
TDTM: Transfusion-dependent thalassemia major
TFS: Thalassemia-free survival
TM: Thalassemia major
