# Supplementary material for: The effect of bone marrow-derived mesenchymal stem cell co-transplantation with hematopoietic stem cells on liver fibrosis alleviation and survival in patients with class III β-thalassemia major
Source: Stem Cell Res Ther. 2021 Mar 29;12:213. doi: 10.1186/s13287-021-02242-8 (PMC8008651; doi:10.1186/s13287-021-02242-8)
Supplement: Supplementary file 1 — Additional file 1. Methodological Appendix. [file 13287_2021_2242_MOESM1_ESM.docx]

**Methodological Appendix**

Mixed-effect regression models investigate changes in Serum ferritin, AST, ALT, FibroScan score, hepatic T2 * MRI, Liver Stage, Liver grade, and liver iron dry weight associated with mesenchymal stem cell intervention injection.

The models were estimated with the Stata xtmixed module using restricted maximum likelihood (REML) for generalized linear mixed models.^1^ REML provides parameter estimates by maximizing the probability that the predicted values agree with the observed data. The approach is iterative, similar to maximum likelihood (ML) estimation, but provides separate estimation for fixed and random effects.

In other words, mixed models are characterized as containing both fixed effects and random effects. The fixed effects are analogous to standard regression coefficients and are estimated directly. The random effects are not directly estimated but are summarized according to their estimated variances and covariances. Although random effects are not directly estimated, you can form the best linear unbiased predictions (BLUPs) of them (and standard errors)^2^ so estimates obtained in this manner are considered preferable.

The following apply to the two models:

- **Dependent variable**: Serum ferritin, AST, ALT, FibroScan score, hepatic T2 * MRI, Liver Stage, and Liver grade.
- **Independent variables**: All models include a time-indexing variable of post-hsct vs. pre-HSCT, dry iron liver score, time between HSCT and intervention, age of patients, a dichotomous indicator of sex matched vs. sex mismatched, a dichotomous indicator of ABO mismatched vs. ABO matched, a dichotomous indicator of aGvHD vs non-aGvHD, a dichotomous indicator of cGvHD vs. non-cGvHD, a dichotomous indicator of intervention of Mesenchyme stem cells injection vs. non-Mesenchyme stem cells injection.
- **Number of observations**: 71 (missing = 6)

**References:**

1. Graubard, B. I., and E. L. Korn. 1996. Modelling the sampling design in the analysis of health surveys. Statistical Methods in Medical Research 5: 263-281.
2. Rabe-Hesketh, S. and Skrondal, A., 2008. Multilevel and longitudinal modeling using Stata. STATA press.

**Table A-1: Model descriptions and fit statistics**

| **Model** | **Brief Description** | **Fit Statistics** | **Brief Interpretation** |
| --- | --- | --- | --- |
|  |  | **log (restricted) likelihood** |  |
| **1** | Main effects model: Changes over time in serum Ferritin status | -1168.2191 | There was a reduction in Ferritin among patients over the time period. |
| **2** | Interaction model: Modification of change over time by Mesenchyme stem cell injection status | -1161.5517 | There was no significant difference in the reduction of Ferritin over the time period when comparing patients who received mesenchyme SC and patients who did not receive mesenchyme SC. |

**Table A-2: Model 1: Change over time** **by serum Ferritin**

| **Independent Variable** | **Coefficient** | **Std error** | **Z-value** | **Pr > \|Z\|** |
| --- | --- | --- | --- | --- |
| **Intercept** | 529.11 | 1151.70 | 0.46 | 0.646 |
| **Time between Intervention and HSCT** | 26.07 | 67.52 | 0.39 | 0.699 |
| **Liver iron dry weight** | 210.51 | 24.71 | 8.52 | <0.0001 |
| **Age** | 31.43 | 49.87 | 0.63 | 0.528 |
| **Sex Matching** |  |  |  |  |
| **Mismatched** | Ref. | Ref. | Ref. | Ref. |
| **Matched** | -477.76 | 459.49 | -1.04 | 0.298 |
| **ABO Mismatching** |  |  |  |  |
| **Matched** | Ref. | Ref. | Ref. | Ref. |
| **Mismatched** | 203.97 | 486.40 | 0.42 | 0.675 |
| **Source of HSCT** |  |  |  |  |
| **BM** | Ref. | Ref. | Ref. | Ref. |
| **PB** | -525.44 | 618.27 | -0.85 | 0.395 |
| **aGvHD** |  |  |  |  |
| **No** | Ref. | Ref. | Ref. | Ref. |
| **Yes** | 65.60 | 492.16 | 0.13 | 0.894 |
| **cGvHD** |  |  |  |  |
| **No** | Ref. | Ref. | Ref. | Ref. |
| **Yes** | 224.13 | 490.36 | 0.46 | 0.648 |
| **Time** |  |  |  |  |
| **Pre-HSCT** | Ref. | Ref. | Ref. | Ref. |
| **Post-HSCT** | -565.34 | 186.11 | -3.04 | 0.002 |
| [**Mesenchymal**](https://www.google.com/search?q=Mesenchymal&spell=1&sa=X&ved=2ahUKEwjH2YfrnJztAhWJITQIHeCbDW4QkeECKAB6BAgSEC8) **stem cell** |  |  |  |  |
| **No** | Ref. | Ref. | Ref. | Ref. |
| **Yes** | -391.00 | 526.16 | -0.74 | 0.457 |

**Table A-3: Model 2: Change over time moderated** **by serum Ferritin**

| **Independent Variable** | **Coefficient** | **Std error** | **Z-value** | **Pr > \|Z\|** |
| --- | --- | --- | --- | --- |
| **Intercept** | 513.68 | 1155.78 | 0.44 | 0.66 |
| **Time between Intervention and HSCT** | 26.12 | 67.53 | 0.39 | 0.70 |
| **Liver iron dry weight** | 210.35 | 24.84 | 8.47 | 0.00 |
| **Age** | 31.46 | 49.87 | 0.63 | 0.53 |
| **Sex Matching** |  |  |  |  |
| **Mismatched** | Ref. | Ref. | Ref. | Ref. |
| **Matched** | -477.87 | 459.53 | -1.04 | 0.30 |
| **ABO Mismatching** |  |  |  |  |
| **Matched** | Ref. | Ref. | Ref. | Ref. |
| **Mismatched** | 204.06 | 486.44 | 0.42 | 0.68 |
| **Source of HSCT** |  |  |  |  |
| **BM** | Ref. | Ref. | Ref. | Ref. |
| **PB** | -525.18 | 618.33 | -0.85 | 0.40 |
| **aGvHD** |  |  |  |  |
| **No** | Ref. | Ref. | Ref. | Ref. |
| **Yes** | 65.82 | 492.21 | 0.13 | 0.89 |
| **cGvHD** |  |  |  |  |
| **No** | Ref. | Ref. | Ref. | Ref. |
| **Yes** | 223.62 | 490.47 | 0.46 | 0.65 |
| **Time** |  |  |  |  |
| **Pre-HSCT** | Ref. | Ref. | Ref. | Ref. |
| **Post-HSCT** | -534.21 | 269.26 | -1.98 | 0.05 |
| [**Mesenchymal**](https://www.google.com/search?q=Mesenchymal&spell=1&sa=X&ved=2ahUKEwjH2YfrnJztAhWJITQIHeCbDW4QkeECKAB6BAgSEC8) **SC** |  |  |  |  |
| **No** | Ref. | Ref. | Ref. | Ref. |
| **Yes** | -365.73 | 550.78 | -0.66 | 0.51 |
| **Time *** [**Mesenchymal**](https://www.google.com/search?q=Mesenchymal&spell=1&sa=X&ved=2ahUKEwjH2YfrnJztAhWJITQIHeCbDW4QkeECKAB6BAgSEC8) **SC** | -49.20 | 310.83 | -0.16 | 0.87 |

**Table A-4: Model 1: Change over time by AST**

| **Independent Variable** | **Coefficient** | **Std error** | **Z-value** | **Pr > \|Z\|** |
| --- | --- | --- | --- | --- |
| **Intercept** | 14.74 | 5.49 | 2.68 | <0.0001 |
| **Time between Intervention and HSCT** | 0.10 | 0.32 | 0.31 | 0.755 |
| **Liver iron dry weight** | 0.18 | 0.16 | 1.18 | 0.239 |
| **Age** | 0.38 | 0.24 | 1.58 | 0.113 |
| **Sex Matching** |  |  |  |  |
| **Mismatched** | Ref. | Ref. | Ref. | Ref. |
| **Matched** | -2.27 | 2.20 | -1.04 | 0.301 |
| **ABO Mismatching** |  |  |  |  |
| **Matched** | Ref. | Ref. | Ref. | Ref. |
| **Mismatched** | 1.08 | 2.32 | 0.47 | 0.641 |
| **Source of HSCT** |  |  |  |  |
| **BM** | Ref. | Ref. | Ref. | Ref. |
| **PB** | -4.16 | 2.95 | -1.41 | 0.159 |
| **aGvHD** |  |  |  |  |
| **No** | Ref. | Ref. | Ref. | Ref. |
| **Yes** | 3.30 | 2.34 | 1.41 | 0.158 |
| **cGvHD** |  |  |  |  |
| **No** | Ref. | Ref. | Ref. | Ref. |
| **Yes** | 0.70 | 2.34 | 0.30 | 0.763 |
| **Time** |  |  |  |  |
| **Pre-HSCT** | Ref. | Ref. | Ref. | Ref. |
| **Post-HSCT** | -0.06 | 1.03 | -0.05 | 0.956 |
| [**Mesenchymal**](https://www.google.com/search?q=Mesenchymal&spell=1&sa=X&ved=2ahUKEwjH2YfrnJztAhWJITQIHeCbDW4QkeECKAB6BAgSEC8) **SC** |  |  |  |  |
| **No** | Ref. | Ref. | Ref. | Ref. |
| **Yes** | 0.36 | 2.52 | 0.14 | 0.885 |

**Table A-5: Model 2: Change over time by AST**

| **Independent Variable** | **Coefficient** | **Std error** | **Z-value** | **Pr > \|Z\|** |
| --- | --- | --- | --- | --- |
| **Intercept** | 15.05 | 5.51 | 2.73 | 0.006 |
| **Time between Intervention and HSCT** | 0.10 | 0.32 | 0.31 | 0.760 |
| **Liver iron dry weight** | 0.19 | 0.16 | 1.19 | 0.233 |
| **Age** | 0.38 | 0.24 | 1.58 | 0.114 |
| **Sex Matching** |  |  |  |  |
| **Mismatched** | Ref. | Ref. | Ref. | Ref. |
| **Matched** | -2.27 | 2.20 | -1.03 | 0.302 |
| **ABO Mismatching** |  |  |  |  |
| **Matched** | Ref. | Ref. | Ref. | Ref. |
| **Mismatched** | 1.07 | 2.31 | 0.46 | 0.643 |
| **Source of HSCT** |  |  |  |  |
| **BM** | Ref. | Ref. | Ref. | Ref. |
| **PB** | -4.16 | 2.95 | -1.41 | 0.158 |
| **aGvHD** |  |  |  |  |
| **No** | Ref. | Ref. | Ref. | Ref. |
| **Yes** | 3.30 | 2.34 | 1.41 | 0.159 |
| **cGvHD** |  |  |  |  |
| **No** | Ref. | Ref. | Ref. | Ref. |
| **Yes** | 0.72 | 2.34 | 0.31 | 0.758 |
| **Time** |  |  |  |  |
| **Pre-HSCT** | Ref. | Ref. | Ref. | Ref. |
| **Post-HSCT** | -0.68 | 1.49 | -0.46 | 0.647 |
| [**Mesenchymal**](https://www.google.com/search?q=Mesenchymal&spell=1&sa=X&ved=2ahUKEwjH2YfrnJztAhWJITQIHeCbDW4QkeECKAB6BAgSEC8) **SC** |  |  |  |  |
| **No** | Ref. | Ref. | Ref. | Ref. |
| **Yes** | -0.16 | 2.67 | -0.06 | 0.952 |
| **Time *** [**Mesenchymal**](https://www.google.com/search?q=Mesenchymal&spell=1&sa=X&ved=2ahUKEwjH2YfrnJztAhWJITQIHeCbDW4QkeECKAB6BAgSEC8) **SC** | 1.01 | 1.72 | 0.59 | 0.555 |

**Table A-6: Model 1: Change over time by ALT**

| **Independent Variable** | **Coefficient** | **Std error** | **Z-value** | **Pr > \|Z\|** |
| --- | --- | --- | --- | --- |
| **Intercept** | 10.61 | 7.27 | 1.46 | 0.144 |
| **Time between Intervention and HSCT** | 0.68 | 0.43 | 1.60 | 0.110 |
| **Liver iron dry weight** | 0.04 | 0.21 | 0.21 | 0.833 |
| **Age** | 0.44 | 0.31 | 1.39 | 0.164 |
| **Sex Matching** |  |  |  |  |
| **Mismatched** | Ref. | Ref. | Ref. | Ref. |
| **Matched** | -3.00 | 2.91 | -1.03 | 0.302 |
| **ABO Mismatching** |  |  |  |  |
| **Matched** | Ref. | Ref. | Ref. | Ref. |
| **Mismatched** | 1.55 | 3.06 | 0.51 | 0.613 |
| **Source of HSCT** |  |  |  |  |
| **BM** | Ref. | Ref. | Ref. | Ref. |
| **PB** | -0.22 | 3.91 | -0.06 | 0.955 |
| **aGvHD** |  |  |  |  |
| **No** | Ref. | Ref. | Ref. | Ref. |
| **Yes** | 3.67 | 3.10 | 1.18 | 0.236 |
| **cGvHD** |  |  |  |  |
| **No** | Ref. | Ref. | Ref. | Ref. |
| **Yes** | -2.51 | 3.10 | -0.81 | 0.417 |
| **Time** |  |  |  |  |
| **Pre-HSCT** | Ref. | Ref. | Ref. | Ref. |
| **Post-HSCT** | 2.26 | 1.41 | 1.59 | 0.111 |
| [**Mesenchymal**](https://www.google.com/search?q=Mesenchymal&spell=1&sa=X&ved=2ahUKEwjH2YfrnJztAhWJITQIHeCbDW4QkeECKAB6BAgSEC8) **SC** |  |  |  |  |
| **No** | Ref. | Ref. | Ref. | Ref. |
| **Yes** | 1.77 | 3.33 | 0.53 | 0.595 |

**Table A-7: Model 2: Change over time by ALT**

| **Independent Variable** | **Coefficient** | **Std error** | **Z-value** | **Pr > \|Z\|** |
| --- | --- | --- | --- | --- |
| **Intercept** | 10.41 | 7.30 | 1.43 | 0.154 |
| **Time between Intervention and HSCT** | 0.68 | 0.43 | 1.60 | 0.110 |
| **Liver iron dry weight** | 0.04 | 0.21 | 0.19 | 0.847 |
| **Age** | 0.44 | 0.31 | 1.39 | 0.164 |
| **Sex Matching** |  |  |  |  |
| **Mismatched** | Ref. | Ref. | Ref. | Ref. |
| **Matched** | -2.99 | 2.90 | -1.03 | 0.303 |
| **ABO Mismatching** |  |  |  |  |
| **Matched** | Ref. | Ref. | Ref. | Ref. |
| **Mismatched** | 1.55 | 3.06 | 0.50 | 0.614 |
| **Source of HSCT** |  |  |  |  |
| **BM** | Ref. | Ref. | Ref. | Ref. |
| **PB** | -0.21 | 3.90 | -0.05 | 0.957 |
| **aGvHD** |  |  |  |  |
| **No** | Ref. | Ref. | Ref. | Ref. |
| **Yes** | 3.67 | 3.10 | 1.18 | 0.236 |
| **cGvHD** |  |  |  |  |
| **No** | Ref. | Ref. | Ref. | Ref. |
| **Yes** | -2.52 | 3.09 | -0.81 | 0.416 |
| **Time** |  |  |  |  |
| **Pre-HSCT** | Ref. | Ref. | Ref. | Ref. |
| **Post-HSCT** | 2.64 | 2.07 | 1.28 | 0.202 |
| [**Mesenchymal**](https://www.google.com/search?q=Mesenchymal&spell=1&sa=X&ved=2ahUKEwjH2YfrnJztAhWJITQIHeCbDW4QkeECKAB6BAgSEC8) **SC** |  |  |  |  |
| **No** | Ref. | Ref. | Ref. | Ref. |
| **Yes** | 2.07 | 3.55 | 0.58 | 0.561 |
| **Time *** [**Mesenchymal**](https://www.google.com/search?q=Mesenchymal&spell=1&sa=X&ved=2ahUKEwjH2YfrnJztAhWJITQIHeCbDW4QkeECKAB6BAgSEC8) | -0.58 | 2.40 | -0.24 | 0.809 |

**Table A-8: Model 1: Change over time by** **Fibroscan score**

| **Independent Variable** | **Coefficient** | **Std error** | **Z-value** | **Pr > \|Z\|** |
| --- | --- | --- | --- | --- |
| **Intercept** | 0.53 | 1.76 | 0.30 | 0.764 |
| **Time between Intervention and HSCT** | 0.03 | 0.10 | 0.30 | 0.760 |
| **Liver iron dry weight** | 0.29 | 0.05 | 5.58 | <0.0001 |
| **Age** | 0.05 | 0.08 | 0.61 | 0.540 |
| **Sex Matching** |  |  |  |  |
| **Mismatched** | Ref. | Ref. | Ref. | Ref. |
| **Matched** | 0.18 | 0.70 | 0.26 | 0.794 |
| **ABO Mismatching** |  |  |  |  |
| **Matched** | Ref. | Ref. | Ref. | Ref. |
| **Mismatched** | 0.78 | 0.74 | 1.06 | 0.291 |
| **Source of HSCT** |  |  |  |  |
| **BM** | Ref. | Ref. | Ref. | Ref. |
| **PB** | 1.20 | 0.95 | 1.27 | 0.205 |
| **aGvHD** |  |  |  |  |
| **No** | Ref. | Ref. | Ref. | Ref. |
| **Yes** | 0.14 | 0.75 | 0.18 | 0.856 |
| **cGvHD** |  |  |  |  |
| **No** | Ref. | Ref. | Ref. | Ref. |
| **Yes** | -0.18 | 0.75 | -0.24 | 0.807 |
| **Time** |  |  |  |  |
| **Pre-HSCT** | Ref. | Ref. | Ref. | Ref. |
| **Post-HSCT** | 1.64 | 0.36 | 4.56 | <0.0001 |
| [**Mesenchymal**](https://www.google.com/search?q=Mesenchymal&spell=1&sa=X&ved=2ahUKEwjH2YfrnJztAhWJITQIHeCbDW4QkeECKAB6BAgSEC8) **SC** |  |  |  |  |
| **No** | Ref. | Ref. | Ref. | Ref. |
| **Yes** | -0.07 | 0.81 | -0.09 | 0.930 |

**Table A-9: Model 2: Change over time by Fibroscan score**

| **Independent Variable** | **Coefficient** | **Std error** | **Z-value** | **Pr > \|Z\|** |
| --- | --- | --- | --- | --- |
| **Intercept** | 0.49 | 1.77 | 0.28 | 0.782 |
| **Time between Intervention and HSCT** | 0.03 | 0.10 | 0.30 | 0.762 |
| **Liver iron dry weight** | 0.29 | 0.05 | 5.56 | <0.0001 |
| **Age** | 0.05 | 0.08 | 0.61 | 0.542 |
| **Sex Matching** |  |  |  |  |
| **Mismatched** | Ref. | Ref. | Ref. | Ref. |
| **Matched** | 0.19 | 0.70 | 0.26 | 0.792 |
| **ABO Mismatching** |  |  |  |  |
| **Matched** | Ref. | Ref. | Ref. | Ref. |
| **Mismatched** | 0.78 | 0.74 | 1.06 | 0.291 |
| **Source of HSCT** |  |  |  |  |
| **BM** | Ref. | Ref. | Ref. | Ref. |
| **PB** | 1.20 | 0.94 | 1.27 | 0.204 |
| **aGvHD** |  |  |  |  |
| **No** | Ref. | Ref. | Ref. | Ref. |
| **Yes** | 0.13 | 0.75 | 0.18 | 0.857 |
| **cGvHD** |  |  |  |  |
| **No** | Ref. | Ref. | Ref. | Ref. |
| **Yes** | -0.18 | 0.75 | -0.24 | 0.808 |
| **Time** |  |  |  |  |
| **Pre-HSCT** | Ref. | Ref. | Ref. | Ref. |
| **Post-HSCT** | 1.71 | 0.53 | 3.21 | 0.001 |
| [**Mesenchymal**](https://www.google.com/search?q=Mesenchymal&spell=1&sa=X&ved=2ahUKEwjH2YfrnJztAhWJITQIHeCbDW4QkeECKAB6BAgSEC8) **SC** |  |  |  |  |
| **No** | Ref. | Ref. | Ref. | Ref. |
| **Yes** | -0.02 | 0.87 | -0.02 | 0.983 |
| **Time *** [**Mesenchymal**](https://www.google.com/search?q=Mesenchymal&spell=1&sa=X&ved=2ahUKEwjH2YfrnJztAhWJITQIHeCbDW4QkeECKAB6BAgSEC8) | -0.10 | 0.62 | -0.17 | 0.866 |

**Table A-10: Model 1: Change over time by Hepatic T2*MRI**

| **Independent Variable** | **Coefficient** | **Std error** | **Z-value** | **Pr > \|Z\|** |
| --- | --- | --- | --- | --- |
| **Intercept** | 23.94 | 6.07 | 3.95 | <0.0001 |
| **Time between Intervention and HSCT** | 0.25 | 0.36 | 0.71 | 0.479 |
| **Liver iron dry weight** | -0.31 | 0.18 | -1.78 | 0.076 |
| **Age** | 0.11 | 0.26 | 0.40 | 0.686 |
| **Sex Matching** |  |  |  |  |
| **Mismatched** | Ref. | Ref. | Ref. | Ref. |
| **Matched** | -2.73 | 2.43 | -1.12 | 0.261 |
| **ABO Mismatching** |  |  |  |  |
| **Matched** | Ref. | Ref. | Ref. | Ref. |
| **Mismatched** | 1.64 | 2.56 | 0.64 | 0.522 |
| **Source of HSCT** |  |  |  |  |
| **BM** | Ref. | Ref. | Ref. | Ref. |
| **PB** | -1.20 | 3.26 | -0.37 | 0.712 |
| **aGvHD** |  |  |  |  |
| **No** | Ref. | Ref. | Ref. | Ref. |
| **Yes** | -1.73 | 2.59 | -0.67 | 0.503 |
| **cGvHD** |  |  |  |  |
| **No** | Ref. | Ref. | Ref. | Ref. |
| **Yes** | 0.98 | 2.59 | 0.38 | 0.705 |
| **Time** |  |  |  |  |
| **Pre-HSCT** | Ref. | Ref. | Ref. | Ref. |
| **Post-HSCT** | 1.92 | 1.18 | 1.63 | 0.102 |
| [**Mesenchymal**](https://www.google.com/search?q=Mesenchymal&spell=1&sa=X&ved=2ahUKEwjH2YfrnJztAhWJITQIHeCbDW4QkeECKAB6BAgSEC8) **SC** |  |  |  |  |
| **No** | Ref. | Ref. | Ref. | Ref. |
| **Yes** | -2.96 | 2.78 | -1.06 | 0.288 |

**Table A-11: Model 2: Change over time by** **Hepatic T2*MRI**

| **Independent Variable** | **Coefficient** | **Std error** | **Z-value** | **Pr > \|Z\|** |
| --- | --- | --- | --- | --- |
| **Intercept** | 23.77 | 6.09 | 3.95 | <0.0001 |
| **Time between Intervention and HSCT** | 0.25 | 0.35 | 0.72 | 0.473 |
| **Liver iron dry weight** | -0.32 | 0.18 | -1.80 | 0.072 |
| **Age** | 0.11 | 0.26 | 0.41 | 0.681 |
| **Sex Matching** |  |  |  |  |
| **Mismatched** | Ref. | Ref. | Ref. | Ref. |
| **Matched** | -2.74 | 2.42 | -1.13 | 0.259 |
| **ABO Mismatching** |  |  |  |  |
| **Matched** | Ref. | Ref. | Ref. | Ref. |
| **Mismatched** | 1.64 | 2.55 | 0.64 | 0.520 |
| **Source of HSCT** |  |  |  |  |
| **BM** | Ref. | Ref. | Ref. | Ref. |
| **PB** | -1.20 | 3.26 | -0.37 | 0.713 |
| **aGvHD** |  |  |  |  |
| **No** | Ref. | Ref. | Ref. | Ref. |
| **Yes** | -1.72 | 2.58 | -0.67 | 0.505 |
| **cGvHD** |  |  |  |  |
| **No** | Ref. | Ref. | Ref. | Ref. |
| **Yes** | 0.96 | 2.58 | 0.37 | 0.711 |
| **Time** |  |  |  |  |
| **Pre-HSCT** | Ref. | Ref. | Ref. | Ref. |
| **Post-HSCT** | 2.30 | 1.72 | 1.33 | 0.182 |
| [**Mesenchymal**](https://www.google.com/search?q=Mesenchymal&spell=1&sa=X&ved=2ahUKEwjH2YfrnJztAhWJITQIHeCbDW4QkeECKAB6BAgSEC8) **SC** |  |  |  |  |
| **No** | Ref. | Ref. | Ref. | Ref. |
| **Yes** | -2.61 | 2.96 | -0.88 | 0.379 |
| **Time *** [**Mesenchymal**](https://www.google.com/search?q=Mesenchymal&spell=1&sa=X&ved=2ahUKEwjH2YfrnJztAhWJITQIHeCbDW4QkeECKAB6BAgSEC8) | -0.65 | 2.00 | -0.32 | 0.746 |

**Table A-12: Model 1: Change over time by Cardiac T2*MRI**

| **Independent Variable** | **Coefficient** | **Std error** | **Z-value** | **Pr > \|Z\|** |
| --- | --- | --- | --- | --- |
| **Intercept** | 27.98 | 3.17 | 8.83 | <0.0001 |
| **Time between Intervention and HSCT** | -0.02 | 0.19 | -0.09 | 0.925 |
| **Liver iron dry weight** | -0.30 | 0.09 | -3.23 | 0.001 |
| **Age** | 0.02 | 0.14 | 0.11 | 0.912 |
| **Sex Matching** |  |  |  |  |
| **Mismatched** | Ref. | Ref. | Ref. | Ref. |
| **Matched** | -0.96 | 1.27 | -0.76 | 0.449 |
| **ABO Mismatching** |  |  |  |  |
| **Matched** | Ref. | Ref. | Ref. | Ref. |
| **Mismatched** | -1.35 | 1.34 | -1.01 | 0.314 |
| **Source of HSCT** |  |  |  |  |
| **BM** | Ref. | Ref. | Ref. | Ref. |
| **PB** | -1.15 | 1.70 | -0.67 | 0.500 |
| **aGvHD** |  |  |  |  |
| **No** | Ref. | Ref. | Ref. | Ref. |
| **Yes** | -0.65 | 1.35 | -0.48 | 0.633 |
| **cGvHD** |  |  |  |  |
| **No** | Ref. | Ref. | Ref. | Ref. |
| **Yes** | 0.36 | 1.35 | 0.27 | 0.790 |
| **Time** |  |  |  |  |
| **Pre-HSCT** | Ref. | Ref. | Ref. | Ref. |
| **Post-HSCT** | 2.30 | 0.61 | 3.80 | <0.0001 |
| [**Mesenchymal**](https://www.google.com/search?q=Mesenchymal&spell=1&sa=X&ved=2ahUKEwjH2YfrnJztAhWJITQIHeCbDW4QkeECKAB6BAgSEC8) **SC** |  |  |  |  |
| **No** | Ref. | Ref. | Ref. | Ref. |
| **Yes** | -1.01 | 1.45 | -0.69 | 0.488 |

**Table A-12: Model 2: Change over time by Cardiac T2*MRI**

| **Independent Variable** | **Coefficient** | **Std error** | **Z-value** | **Pr > \|Z\|** |
| --- | --- | --- | --- | --- |
| **Intercept** | 27.79 | 3.18 | 8.73 | <0.0001 |
| **Time between Intervention and HSCT** | -0.02 | 0.19 | -0.09 | 0.930 |
| **Liver iron dry weight** | -0.30 | 0.09 | -3.25 | 0.001 |
| **Age** | 0.02 | 0.14 | 0.12 | 0.908 |
| **Sex Matching** |  |  |  |  |
| **Mismatched** | Ref. | Ref. | Ref. | Ref. |
| **Matched** | -0.96 | 1.27 | -0.76 | 0.446 |
| **ABO Mismatching** |  |  |  |  |
| **Matched** | Ref. | Ref. | Ref. | Ref. |
| **Mismatched** | -1.34 | 1.34 | -1.01 | 0.315 |
| **Source of HSCT** |  |  |  |  |
| **BM** | Ref. | Ref. | Ref. | Ref. |
| **PB** | -1.15 | 1.70 | -0.67 | 0.501 |
| **aGvHD** |  |  |  |  |
| **No** | Ref. | Ref. | Ref. | Ref. |
| **Yes** | -0.64 | 1.35 | -0.47 | 0.635 |
| **cGvHD** |  |  |  |  |
| **No** | Ref. | Ref. | Ref. | Ref. |
| **Yes** | 0.35 | 1.35 | 0.26 | 0.795 |
| **Time** |  |  |  |  |
| **Pre-HSCT** | Ref. | Ref. | Ref. | Ref. |
| **Post-HSCT** | 2.70 | 0.88 | 3.06 | 0.002 |
| [**Mesenchymal**](https://www.google.com/search?q=Mesenchymal&spell=1&sa=X&ved=2ahUKEwjH2YfrnJztAhWJITQIHeCbDW4QkeECKAB6BAgSEC8) **SC** |  |  |  |  |
| **No** | Ref. | Ref. | Ref. | Ref. |
| **Yes** | -0.68 | 1.54 | -0.44 | 0.661 |
| **Time *** [**Mesenchymal**](https://www.google.com/search?q=Mesenchymal&spell=1&sa=X&ved=2ahUKEwjH2YfrnJztAhWJITQIHeCbDW4QkeECKAB6BAgSEC8) | -0.64 | 1.02 | -0.62 | 0.534 |

**Table A-13: Model 1: Change over time by liver stage**

| **Independent Variable** | **Coefficient** | **Std error** | **Z-value** | **Pr > \|Z\|** |
| --- | --- | --- | --- | --- |
| **Intercept** | 0.20 | 0.46 | 0.42 | 0.673 |
| **Time between Intervention and HSCT** | 0.05 | 0.03 | 1.73 | 0.083 |
| **Liver iron dry weight** | 0.06 | 0.02 | 3.92 | <0.0001 |
| **Age** | 0.02 | 0.02 | 1.11 | 0.268 |
| **Sex Matching** |  |  |  |  |
| **Mismatched** | Ref. | Ref. | Ref. | Ref. |
| **Matched** | 0.04 | 0.18 | 0.22 | 0.827 |
| **ABO Mismatching** |  |  |  |  |
| **Matched** | Ref. | Ref. | Ref. | Ref. |
| **Mismatched** | 0.05 | 0.19 | 0.27 | 0.785 |
| **Source of HSCT** |  |  |  |  |
| **BM** | Ref. | Ref. | Ref. | Ref. |
| **PB** | -0.24 | 0.25 | -0.98 | 0.328 |
| **aGvHD** |  |  |  |  |
| **No** | Ref. | Ref. | Ref. | Ref. |
| **Yes** | 0.22 | 0.20 | 1.14 | 0.254 |
| **cGvHD** |  |  |  |  |
| **No** | Ref. | Ref. | Ref. | Ref. |
| **Yes** | -0.15 | 0.20 | -0.77 | 0.440 |
| **Time** |  |  |  |  |
| **Pre-HSCT** | Ref. | Ref. | Ref. | Ref. |
| **Post-HSCT** | -0.17 | 0.12 | -1.42 | 0.156 |
| [**Mesenchymal**](https://www.google.com/search?q=Mesenchymal&spell=1&sa=X&ved=2ahUKEwjH2YfrnJztAhWJITQIHeCbDW4QkeECKAB6BAgSEC8) **SC** |  |  |  |  |
| **No** | Ref. | Ref. | Ref. | Ref. |
| **Yes** | -0.12 | 0.21 | -0.59 | 0.558 |

**Table A-13: Model 2: Change over time by liver stage**

| **Independent Variable** | **Coefficient** | **Std error** | **Z-value** | **Pr > \|Z\|** |
| --- | --- | --- | --- | --- |
| **Intercept** | 0.24 | 0.47 | 0.52 | 0.602 |
| **Time between Intervention and HSCT** | 0.05 | 0.03 | 1.73 | 0.084 |
| **Liver iron dry weight** | 0.06 | 0.02 | 3.93 | <0.0001 |
| **Age** | 0.02 | 0.02 | 1.10 | 0.270 |
| **Sex Matching** |  |  |  |  |
| **Mismatched** | Ref. | Ref. | Ref. | Ref. |
| **Matched** | 0.04 | 0.18 | 0.22 | 0.825 |
| **ABO Mismatching** |  |  |  |  |
| **Matched** | Ref. | Ref. | Ref. | Ref. |
| **Mismatched** | 0.05 | 0.19 | 0.27 | 0.786 |
| **Source of HSCT** |  |  |  |  |
| **BM** | Ref. | Ref. | Ref. | Ref. |
| **PB** | -0.24 | 0.25 | -0.98 | 0.327 |
| **aGvHD** |  |  |  |  |
| **No** | Ref. | Ref. | Ref. | Ref. |
| **Yes** | 0.22 | 0.20 | 1.14 | 0.255 |
| **cGvHD** |  |  |  |  |
| **No** | Ref. | Ref. | Ref. | Ref. |
| **Yes** | -0.15 | 0.20 | -0.77 | 0.443 |
| **Time** |  |  |  |  |
| **Pre-HSCT** | Ref. | Ref. | Ref. | Ref. |
| **Post-HSCT** | -0.27 | 0.19 | -1.43 | 0.152 |
| [**Mesenchymal**](https://www.google.com/search?q=Mesenchymal&spell=1&sa=X&ved=2ahUKEwjH2YfrnJztAhWJITQIHeCbDW4QkeECKAB6BAgSEC8) **SC** |  |  |  |  |
| **No** | Ref. | Ref. | Ref. | Ref. |
| **Yes** | -0.20 | 0.24 | -0.84 | 0.401 |
| **Time * Mesenchymal** | 0.16 | 0.23 | 0.68 | 0.497 |

**Table A-14: Model 1: Change over time by liver grade**

| **Independent Variable** | **Coefficient** | **Std error** | **Z-value** | **Pr > \|Z\|** |
| --- | --- | --- | --- | --- |
| **Intercept** | 0.33 | 0.52 | 0.64 | 0.524 |
| **Time between Intervention and HSCT** | 0.01 | 0.03 | 0.29 | 0.772 |
| **Liver iron dry weight** | 0.16 | 0.02 | 8.87 | <0.0001 |
| **Age** | 0.02 | 0.02 | 0.93 | 0.353 |
| **Sex Matching** |  |  |  |  |
| **Mismatched** | Ref. | Ref. | Ref. | Ref. |
| **Matched** | -0.27 | 0.21 | -1.30 | 0.195 |
| **ABO Mismatching** |  |  |  |  |
| **Matched** | Ref. | Ref. | Ref. | Ref. |
| **Mismatched** | -0.18 | 0.22 | -0.81 | 0.418 |
| **Source of HSCT** |  |  |  |  |
| **BM** | Ref. | Ref. | Ref. | Ref. |
| **PB** | -0.03 | 0.28 | -0.11 | 0.910 |
| **aGvHD** |  |  |  |  |
| **No** | Ref. | Ref. | Ref. | Ref. |
| **Yes** | 0.22 | 0.22 | 0.99 | 0.323 |
| **cGvHD** |  |  |  |  |
| **No** | Ref. | Ref. | Ref. | Ref. |
| **Yes** | -0.13 | 0.22 | -0.57 | 0.566 |
| **Time** |  |  |  |  |
| **Pre-HSCT** | Ref. | Ref. | Ref. | Ref. |
| **Post-HSCT** | 0.15 | 0.18 | 0.85 | 0.397 |
| [**Mesenchymal**](https://www.google.com/search?q=Mesenchymal&spell=1&sa=X&ved=2ahUKEwjH2YfrnJztAhWJITQIHeCbDW4QkeECKAB6BAgSEC8) **SC** |  |  |  |  |
| **No** | Ref. | Ref. | Ref. | Ref. |
| **Yes** | 0.02 | 0.24 | 0.07 | 0.941 |

**Table A-15: Model 2: Change over time by liver grade**

| **Independent Variable** | **Coefficient** | **Std error** | **Z-value** | **Pr > \|Z\|** |
| --- | --- | --- | --- | --- |
| **Intercept** | 0.38 | 0.54 | 0.71 | 0.479 |
| **Time between Intervention and HSCT** | 0.01 | 0.03 | 0.29 | 0.774 |
| **Liver iron dry weight** | 0.16 | 0.02 | 8.88 | <0.0001 |
| **Age** | 0.02 | 0.02 | 0.93 | 0.355 |
| **Sex Matching** |  |  |  |  |
| **Mismatched** | Ref. | Ref. | Ref. | Ref. |
| **Matched** | -0.27 | 0.21 | -1.29 | 0.195 |
| **ABO Mismatching** |  |  |  |  |
| **Matched** | Ref. | Ref. | Ref. | Ref. |
| **Mismatched** | -0.18 | 0.22 | -0.81 | 0.417 |
| **Source of HSCT** |  |  |  |  |
| **BM** | Ref. | Ref. | Ref. | Ref. |
| **PB** | -0.03 | 0.28 | -0.11 | 0.910 |
| **aGvHD** |  |  |  |  |
| **No** | Ref. | Ref. | Ref. | Ref. |
| **Yes** | 0.22 | 0.22 | 0.99 | 0.324 |
| **cGvHD** |  |  |  |  |
| **No** | Ref. | Ref. | Ref. | Ref. |
| **Yes** | -0.13 | 0.22 | -0.57 | 0.569 |
| **Time** |  |  |  |  |
| **Pre-HSCT** | Ref. | Ref. | Ref. | Ref. |
| **Post-HSCT** | 0.06 | 0.29 | 0.21 | 0.835 |
| [**Mesenchymal**](https://www.google.com/search?q=Mesenchymal&spell=1&sa=X&ved=2ahUKEwjH2YfrnJztAhWJITQIHeCbDW4QkeECKAB6BAgSEC8) **SC** |  |  |  |  |
| **No** | Ref. | Ref. | Ref. | Ref. |
| **Yes** | -0.06 | 0.30 | -0.19 | 0.852 |
| **Time * Mesenchymal** | 0.14 | 0.35 | 0.41 | 0.680 |

**Table A-16: Model 1: Change over time by Liver iron dry weight**

| **Independent Variable** | **Coefficient** | **Std error** | **Z-value** | **Pr > \|Z\|** |
| --- | --- | --- | --- | --- |
| **Intercept** | 1.17 | 3.48 | 0.34 | 0.737 |
| **Time between Intervention and HSCT** | 0.16 | 0.20 | 0.79 | 0.429 |
| **Age** | 0.06 | 0.15 | 0.38 | 0.705 |
| **Sex Matching** |  |  |  |  |
| **Mismatched** | Ref. | Ref. | Ref. | Ref. |
| **Matched** | 0.34 | 1.39 | 0.24 | 0.808 |
| **ABO Mismatching** |  |  |  |  |
| **Matched** | Ref. | Ref. | Ref. | Ref. |
| **Mismatched** | 0.03 | 1.47 | 0.02 | 0.984 |
| **Source of HSCT** |  |  |  |  |
| **BM** | Ref. | Ref. | Ref. | Ref. |
| **PB** | 1.93 | 1.86 | 1.04 | 0.301 |
| **aGvHD** |  |  |  |  |
| **No** | Ref. | Ref. | Ref. | Ref. |
| **Yes** | 0.78 | 1.48 | 0.52 | 0.600 |
| **cGvHD** |  |  |  |  |
| **No** | Ref. | Ref. | Ref. | Ref. |
| **Yes** | -2.45 | 1.46 | -1.67 | 0.094 |
| **Time** |  |  |  |  |
| **Pre-HSCT** | Ref. | Ref. | Ref. | Ref. |
| **Post-HSCT** | -2.08 | 0.50 | -4.18 | <0.0001 |
| [**Mesenchymal**](https://www.google.com/search?q=Mesenchymal&spell=1&sa=X&ved=2ahUKEwjH2YfrnJztAhWJITQIHeCbDW4QkeECKAB6BAgSEC8) **SC** |  |  |  |  |
| **No** | Ref. | Ref. | Ref. | Ref. |
| **Yes** | 3.40 | 1.56 | 2.18 | 0.030 |

**Table A-17: Model 2: Change over time by Liver iron dry weight**

| **Independent Variable** | **Coefficient** | **Std error** | **Z-value** | **Pr > \|Z\|** |
| --- | --- | --- | --- | --- |
| **Intercept** | 1.02 | 3.49 | 0.29 | 0.770 |
| **Time between Intervention and HSCT** | 0.16 | 0.20 | 0.79 | 0.428 |
| **Age** | 0.06 | 0.15 | 0.38 | 0.703 |
| **Sex Matching** |  |  |  |  |
| **Mismatched** | Ref. | Ref. | Ref. | Ref. |
| **Matched** | 0.33 | 1.39 | 0.24 | 0.810 |
| **ABO Mismatching** |  |  |  |  |
| **Matched** | Ref. | Ref. | Ref. | Ref. |
| **Mismatched** | 0.03 | 1.47 | 0.02 | 0.983 |
| **Source of HSCT** |  |  |  |  |
| **BM** | Ref. | Ref. | Ref. | Ref. |
| **PB** | 1.93 | 1.86 | 1.03 | 0.301 |
| **aGvHD** |  |  |  |  |
| **No** | Ref. | Ref. | Ref. | Ref. |
| **Yes** | 0.78 | 1.49 | 0.53 | 0.600 |
| **cGvHD** |  |  |  |  |
| **No** | Ref. | Ref. | Ref. | Ref. |
| **Yes** | -2.45 | 1.46 | -1.67 | 0.094 |
| **Time** |  |  |  |  |
| **Pre-HSCT** | Ref. | Ref. | Ref. | Ref. |
| **Post-HSCT** | -1.78 | 0.72 | -2.47 | 0.013 |
| [**Mesenchymal**](https://www.google.com/search?q=Mesenchymal&spell=1&sa=X&ved=2ahUKEwjH2YfrnJztAhWJITQIHeCbDW4QkeECKAB6BAgSEC8) **SC** |  |  |  |  |
| **No** | Ref. | Ref. | Ref. | Ref. |
| **Yes** | 3.64 | 1.62 | 2.25 | 0.024 |
| **Time * Mesenchymal** | -0.47 | 0.83 | -0.57 | 0.566 |
